# Functions of Autobiographical Memory in Younger and Older Adults

**DOI:** 10.3389/fpsyg.2018.00219

**Published:** 2018-02-27

**Authors:** Andrea Vranić, Margareta Jelić, Mirjana Tonković

**Affiliations:** Department of Psychology, University of Zagreb, Zagreb, Croatia

**Keywords:** autobiographical memory, TALE scale, validation study, confirmatory factor analysis, age differences, autobiographical memory functions

## Abstract

Functional approach to autobiographical memory (AM) posits its three broad functions: directive, self, and social. Although these functions are probably universal, life stage and gender variations are expected. This research builds on previous studies investigating the validity of Thinking About Life Experiences Questionnaire (TALE; Bluck and Alea, 2011). A sample of 365 adults (56% female, mean age 43.3 years), divided in 2 age cohorts (young: 18–45 years, old: 46–90 years), used TALE, to rate their tendency of using AM for three different purposes, and measures of self-concept clarity, attachment in close relationships and time perspective. Confirmatory factor analysis of TALE confirmed the tripartite model of AM functions and further analysis showed partial factorial equivalence across age and gender groups. Young tend to use AM more for directing future behavior and social-bonding, while no age differences were found in the use of AM to serve self-function. As for gender variations, women tend to use AM more for directing their behavior, while no other gender differences in the use of AM were found. TALE showed good internal consistency and convergent validity of the three subscales. The theory-driven hypotheses that individuals with low self-concept clarity would use AM more often to serve a self-function, those with higher levels of attachment anxiety would use AM more often to serve a social function, and those past-oriented would use memory more often for directive purpose, were all confirmed. Also confirmed was the notion of Past Negative Orientation to be more related to the directive use of AM than Past Positive Time Orientation. Limitations and future directions are discussed.

## Introduction

Autobiographical memory (AM) research is mostly focused on the quantity and accuracy of remembering. In comparison to understanding how AM works, a literature on why we remember personal life events over long periods is relatively understudied. From the theoretical point of view, a number of ideas on the functions of AM have been put forth (Graumann, 1986; Neisser, 1988), yet not as often have these ideas been empirically validated. When tapping into the functionality of AM we discuss either its use, or its adaptivity (Bluck and Alea, 2002; Pillemer, 2009). Although the use and adaptivity of memory are most certainly intertwined, the appropriate research direction seems to be: (1) to identify the ways in which AM is used, and (2) to elaborate on its (mal)adaptivity in everyday life. This idea is mirrored in the recent advancements in AM research resulting in theory-based questionnaires of AM functions (e.g., Thinking of Life Experience Scale—TALE; (Bluck et al., 2005); “Things about memory” questionnaire, Wang et al., 2014). Three basic theory-driven functions of AM, nested in these scales, are directive (planning for present and future behaviors), self (self-continuity, self-expression), and social (communication, relationship maintenance) function (Pillemer, 1992; Bluck and Alea, 2002; Bluck et al., 2005). Although AM may serve other functions beyond those suggested by the tripartite model (e.g., Webster, 1993; Pasupathi, 2003; Harris et al., 2014), most researchers agree upon the three major functions (Bluck, 2003; Pillemer, 2003).

### The directive function

This function of AM describes the use of past experiences to direct or guide current and future actions, thoughts and behaviors. In so far, studies have outlined numerous ways in which directive nature of AM is evident. Information stored in AM supports everyday decision making by retrieving past experiences to enable solving of current problems and predict future events (Baddeley, 1988; Bluck et al., 2005). Robinson and Swanson (1990) take this assumption a step further by suggesting that AM helps us create models of encountered events and people which allow better understanding of other's inner worlds. Such models also facilitate prediction of other people's behavior. Cohen (1998) complements these assumptions by arguing that AM can also lead to the development of personal opinions and attitudes. Empirical data fits well with these ideas: personal reports about reminiscence of past events are inwrought with morals on importance of lessons learnt for our future behavior (McCabe et al., 1991; Pratt et al., 1999; Bluck and Glück, 2004).

### The self function

AM serves as a repository of self-information that play a critical role in creating stable and enduring representation of our selves over time. The self-function refers to the use of personal information to maintain a sense of being the same person during one's lifetime or to update the self while maintaining continuity (Barclay, 1996; Conway, 2003). Autobiographical narratives jointly form a stable identity, beginning in adolescence (McAdams, 1995; Habermas and Bluck, 2000), and continuing throughout the life span (Singer and Blagov, 2004; Waters et al., 2014). This sense of identity is then conveyed to others through expressing and sharing of AM. Overall, knowledge of the self in the past and its echo projected into the future is viewed as a critical type of self-knowledge (Neisser, 1988; Bluck and Alea, 2008) and its adequacy depends on its capacity to reinforce continuity and development of the self. Empirical work finds support for self-function—Bluck and Alea (2008) found that people with low self-concept clarity (i.e., with poorly defined and internally inconsistent self-concept) are more likely to use and recall the past for self-continuity purposes, suggesting that memories can be used to “remind” an individual of the sense of identity.

### The social function

Several authors have argued that social bonding via retrieving and sharing of personal memories is the primary function of AM (Neisser, 1978; Nelson, 1993). Autobiographical memories are viewed as vignettes used to initiate, develop, foster and maintain social bonds and relations (Nelson, 1993, 2003; Pillemer, 1998). Most commonly, personal memories serve as a medium for sharing of experiences, thus facilitating understanding and empathy in social interactions (Alea and Bluck, 2003). Sharing of AM may foster intimacy function in regard to the conversational partner because people tend to feel closer to the listener after sharing an AM due to self-disclosure processes (Laurenceau et al., 1998; Alea and Bluck, 2007). However, AM may involve memories about loved ones, which are not necessarily shared with others (Alea and Bluck, 2007; Kulkofsky et al., 2010). The social function obviously serves a myriad of purposes. It is probably best viewed as an umbrella dimension, which is differentiated into specific, yet complementing, sub-functions. Empirical studies are granting proofs for such a perspective. For example, the use of AM to pass on valuable life lessons, the so-called Teach/Inform function, has also been suggested as a part of the AM's social function (Webster, 1993). Also, in the initial analysis of Bluck et al. (2005), social function was split into two factors addressing the nurturing of existent relationships and development of new ones, although authors have argued that these two functions are a reflection of different phases of the connections across generations (Rasmussen and Habermas, 2011).

Functional approach to memory officially began with Baddeley's (1988) article that prompted researchers to examine memory phenomena in everyday context. Almost 30 years later, a number of active scholars has firmly grounded their research in functional perspective (e.g., Nelson, 1993; Webster, 1993; Fivush, 1998; Pillemer, 1998; Alea and Bluck, 2007; Baddeley and Singer, 2008; Rasmussen and Berntsen, 2009; Kulkofsky et al., 2010). Empirical studies derived from these theoretical views are still relatively scarce due to the lack of a standard measurement tool. The most prominent leap in this research sector is probably evident in the construction and later psychometric fine-tuning and validation of the TALE questionnaire (Bluck et al., 2005; Bluck and Alea, 2009, 2011; Rasmussen and Habermas, 2011; Wolf and Zimprich, 2015). The development of TALE begun with an exploratory factor analysis as a preliminary analysis, followed by confirmatory factor analysis (Bluck et al., 2005), and resulted in a brief 15-item self-report measure tapping into the three major functions of AM.

The TALE features items with high face validity and studies have shown good internal consistency for each subscale (Bluck and Alea, 2011). Empirical research assessing convergent and discriminant validity of each of its subscales showed satisfactory results. Bluck and Alea (2011) administered TALE to 156 young and 150 older adults exhibiting typical age differences in basic cognitive functions. Moreover, subset of these participants completed the Self-Concept Clarity Scale, the Future Orientation Scale and the Big Five Inventory—subscales of Extroversion and Neuroticism, in an attempt to provide evidence of validity for self, directive and social functions of AM, respectively. As expected, this study showed good convergent validity. Significant correlations found for the TALE *Self function* and SCSS showed that people with lower self-concept clarity use AM more frequently in an attempt to achieve a clearer self-concept. In terms of discriminant validity the TALE *Self function* did not correlate with personality, or future orientation. Furthermore, the frequency of use of one's past to serve as *Social-bonding function* was found to be related to higher extroversion and did not relate to neither self-concept clarity, nor future orientation. Finally, the *Directive function* subscale was significantly correlated with future orientation indicating that people with a more relaxed view of the future report using AM more to direct their current and future behavior. There was no relation found between Directive subscale and other unrelated constructs (self-concept clarity, extraversion, neuroticism). Also, studies have shown that TALE can be used as a psychologically meaningful tool for examining adult age differences in the functional use of AM (Bluck and Alea, 2008, 2009; Wolf and Zimprich, 2015).

#### The aim

The degree to which certain findings can be generalized is dependent on the generalizability of methodology, thus cross-cultural research is necessary to further validate the TALE (Alea and Wang, 2015). Insofar, metric qualities of the TALE have been investigated in young and older Americans (Bluck and Alea, 2011), Trinidadians (Alea et al., 2015), young Danes, Germans (Rasmussen and Habermas, 2011; Harris et al., 2014), and Japanese (Maki et al., 2015). However, Bluck and Alea (2011) call for further examination of the TALE factor structure in independent samples of older and younger women and men. Studying memory functions in diverse cultural contexts, as well as within-cultural differences (Sahin-Acar and Leichtman, 2015), contributes to our understanding of universality and cultural variations in the everyday use of AM (Alea and Wang, 2015).

This study was conducted with the aim of further investigating the utility of TALE as a valid measure of AM functions, as well as the universality/variability of the use of AM in different cultures. Specific aims of this study were: (i) to further examine construct and discriminant validity of TALE in independent samples of young and older adults, and (ii) to examine adult age and gender differences/similarities in using AM to serve self, social and directive functions. Therefore, the TALE was administered to a sample of younger and older adults. In addition, to assess convergent and discriminant validity for the three TALE subscales separately, we administered measures of Self-concept clarity (Campbell et al., 1996), Experiences in close relationships (Kamenov and Jeliić, 2003), and Time perspective (Zimbardo and Boyd, 1999), respectively. The hypothesized relations between three TALE subscales and related constructs are shown in Table 1.

**Table 1:** Hypothesized Relations between TALE subscales (self-continuity, social bonding, directing behavior) and related constructs.

**Measure**	**Self-continuity**	**Social bonding**	**Directing behavior**
Self-clarity	✓✓	?	?
ECR-R avoidance	?	None	None
ECR-R anxiety	?	✓✓	None
ZTPI past-positive	?	?	✓
ZTPI past-negative	?	?	✓✓

*✓, significant positive correlation expected. ✓✓, significant positive correlation highly expected. ?, unsure about the relation expected. None, no significant correlation expected*.

We expected that individuals with low self-concept clarity would use memory more often to serve a self-function. Furthermore, we hypothesized that individuals with higher levels of attachment anxiety (i.e., those worried about the quality and stability of their relationship) might use memory more often to serve a social function. By remembering past events and sharing them with others (especially their romantic partner) they aim to build stronger bond with relevant others. On the other hand, we expected that avoidant attachment would bear no correlation with social bonding function of AM because people who are uncomfortable being close to others can be motivated either to reduce their discomfort or to dismiss others (depending on their attachment-related anxiety). Moreover, when using AM for social bonding people usually remember positive events, and research confirms that positive memories generally serve social function (Pasupathi et al., 2002; McLean and Lilgendahl, 2008; Rasmussen and Berntsen, 2010). This is hardly surprising because in order to accomplish social bonding, people usually reminisce on the good old days with their friends and share common memories. Avoidant attachment encompasses negative model of others and attachment avoidance is related to recalling more negative memories (Haggerty et al., 2010), as well as quicker retrieval of negative events from the past (He et al., 2011), which does not serve social bonding. Finally, individuals prone to past time orientation should use memory more often for directive purpose. We believed it was likely that negative past orientation will be more related to the directive use of AM than positive past orientation because people are more likely to use their memories in order to avoid repeating the same mistakes in life than to dwell on the past enjoyments when having to make decisions on their future behavior. As for age differences we expected to confirm the results of Bluck and Alea (2009)—younger adults report using AM more for directing behavior and self-continuity purposes, while no differences were expected in the use of AM for social-bonding purposes. Furthermore, Bluck and Alea (2009) report no gender differences in the use of AM.

## Method

### Participants

A sample of 365 adults (56% female, mean age 43.3 years), divided in 2 age cohorts (young: 18–45 years, 56.3% female, mean age 28.7; old: 46–90 years, 54.8% female, mean age 60.36 years), participated in the study (Table 1). Participants were recruited via snowball sampling by graduate psychology students (*N* = 10) who were compensated with research credit for recruiting participants of 2 age cohorts. Participants were not compensated for participation. This study was conducted in accordance with the recommendations of the Ethical code of the Croatian psychological chamber with written informed consent from all subjects. All subjects gave written informed consent in accordance with the Declaration of Helsinki. The protocol was approved by the Ethical committee of the Department of Psychology, Faculty of Humanities and Social Sciences, Zagreb.

### Procedure and measures

Questionnaires were administered in participants' homes. Participants were visited by psychology students and given the informed consent form and the questionnaires. All participants answered the questionnaires by themselves. First, participants completed the 15-item Thinking About Life Experiences scale (TALE; Bluck and Alea, 2011). In order to examine the relation between TALE subscales and other relevant psychological constructs, participants also completed the Self-Concept Clarity Scale (SCCS; Campbell et al., 1996), Shortened version of Brennan's The Experiences in close relationships inventory (ECR-R; Kamenov and Jeliić, 2003), and Zimbardo time perspective inventory (Zimbardo and Boyd, 1999), respectively. All scales were translated and back-translated.

#### Thinking about life experiences scale (TALE)

The Thinking About Life Experiences scale is a 15-item scale assessing the theoretical self, social, and directive functions of AM. The TALE has been translated from English through a standard translation and back-translation procedure. Initial instruction informs participants about autobiographical reasoning (Habermas and Bluck, 2000), i.e., participants are told that thinking and talking about recent and distant memories is an everyday occurrence, and that researchers are interested in how people connect and bring together different events and periods in their life, with no interest in their memory of particular events. First, two baseline questions are presented to assess people's overall tendency to think back or talk about their life. Responses are given on a 5-point Likert-type scale, with 1 = *almost never* to 5 = *very frequently*. Subsequently, and using the same scale, participants indicate how often does their talking or thinking about past events serve a variety of functions. Each item begins with “*I think back over or talk about my life or certain periods of my life…*.” These stem completion items, assessing self-continuity, social and directive function of AM, are presented in mixed order. Each subscale showed high reliability (Cronbach alpha) in this study −0.80 for self-continuity, 0.81 for social and 0.79 for directive subscale.

#### Self-concept clarity scale (SCCS)

The SCCS (Campbell et al., 1996) is a 12-item self-report measure assessing the extent to which the participant's self-concept is clearly and confidently defined, internally consistent and stable. SCCS items direct people to think about the level of clarity and consistency in their view of themselves (e.g., “*It is often hard for me to make up my mind about things because I don't really know what I want*”). Responses are given on a 5-point Likert-type scale, with 1 = *strongly disagree* to 5 = *strongly agree*. Cronbach's alpha in this study was 0.86.

#### Shortened version of brennan's the experiences in close relationships inventory (ECR-R)

The ECR-R (Kamenov and Jeliić, 2003) is a shortened version of the Experiences in Close Relationships Inventory (Brennan et al., 1998) retaining the same psychometric characteristics as the original scale. The scale consists of 18 statements describing one's feelings, thoughts and behaviors in relationship with their romantic partners. The scale consists of two subscales, measuring two attachment dimensions - Anxiety and Avoidance. Anxiety dimension assesses attachment-related anxiety (i.e., how much people are insecure vs. secure about the extent to which their partners are available and responsive) and Avoidance dimension measures attachment-related avoidance (i.e., how much people are uncomfortable being close to others vs. secure depending on others). Each subscale consists of 9 items. The assessments are given on a 7-point scale, ranging from “strongly disagree” to “strongly agree.” Cronbach's alpha in this study was 0.84 for avoidance and 0.80 for anxiety subscale.

#### Zimbardo time perspective inventory (ZTPI)

The ZTPI (Zimbardo and Boyd, 1999) is a 56-item measure with items representing statements about one's beliefs, preferences and values regarding experiences that are temporally based but are not revealing of time related demographics (e.g., “*I've made mistakes in the past that I wish I could undo*”). Respondents are asked to indicate how characteristic a statement is of them on a 5-point Likert-type scale (1 = v*ery uncharacteristic* to 5 = *very characteristic*). Time perspective is a cognitive temporal bias of being past, future or present oriented, which results in a dispositional style that influences one's reaction across a host of everyday life choices (Zimbardo and Boyd, 1999). The ZTPI consists of five subscales: Past-Negative, Past-Positive, Present-Hedonistic, Present-Fatalistic, and Future. Only the Past-Negative and Past-Positive subscales were used in the current study, with Cronbach's alpha being 0.83 and 0.69, respectively. These subscales were included for they reflect a past-oriented temporal bias reflective of using past life-episodes in guiding one's reaction in present and future choices. The Past-Negative orientation reflects a generally negative, aversive view of the past (e.g., “*I think about the good things that I have missed out on in my life*”). The Past-Positive orientation reflects a warm, sentimental attitude toward the past (e.g., “*I like family rituals and traditions that are regularly repeated*”).

### Data analysis

As a first step in the further validation of TALE in Croatian sample, we conducted the CFA. All models were specified and all variance-covariance matrices were analyzed using the *lavaan* package (Rosseel, 2012) in the *R* language and environment (R Development Core Team, 2008). A series of tests of multivariate normality were performed to determine the adequate estimator to be used (since raw items were used as indicators for the measurement models). Mardia's test showed that both multivariate skewness ($\chi_{1,\text{p}}^{2}$ = 23.15, *p* < 0.001) and kurtosis ($\chi_{1,\text{p}}^{2}$ = 310.01, *p* < 0.001) showed a significant departure from normality. This finding corroborated the results of Henze-Zirkler's (*HZ* = 1.12, *p* < 0.001) and Royston's multivariate normality tests (*H* = 896.12, *p* < 0.001). Consequently, we opted for the maximum likelihood with robust (Huber-White) standard errors and a scaled test statistic (Yuan-Bentler). This approach combines the classical ML estimates with the test statistic corrected for departures from normality (Yuan and Bentler, 2000) and robust standard errors (Huber, 1967; White, 1980).

Goodness of fit was evaluated using the aforementioned robust chi-square test, normed chi-square (NC; χ^2^/df), root-mean-square error of approximation (RMSEA) and comparative fit index (CFI). All indicators were calculated based on the robust test statistic. Nested models were compared using the chi-square difference tests (modified for robust test statistic; Satorra and Bentler, 2001). Chi-square *p*-values larger than 0.05, NC below 2, RMSEA below 0.05 and CFI above 0.95 indicated good fit, while NC below 3, RMSEA below 0.08 and CFI above 0.9 indicated adequate fit. To circumvent the problem of missing data full information maximum likelihood (FIML) was used to estimate parameters based on all available information.

## Results

Descriptive statistics for each subscale of TALE in 4 subsamples and the overall sample are shown in Table 2.

**Table 2:** Means (and standard deviations) for each subscale of TALE by age and gender.

	**Male**	**Female**	**Young**	**Old**	**Total**
Self-continuity	2.44 (0.738)	2.63 (0.757)	2.59 (0.743)	2.48 (0.763)	2.54 (0.753)
Social-bonding	2.77 (0.760)	2.85 (0.793)	3.01 (0.746)	2.60 (0.759)	2.81 (0.778)
Directing-behavior	3.04 (0.689)	3.20 (0.721)	3.26 (0.693)	2.97 (0.702)	3.13 (0.711)

In order to find the model that best fits the data several theoretical models were compared, and a final revised model was specified from the best fitting one. Selected information and fit statistics for the models specified are shown in Table 3.

**Table 3:** Comparison of the three alternative models for the three functions of AM.

		**Chi-sq**	***df***	***p***	**NC**	**RMSEA**	**RMSEA 90% CI**	**CFI**	**Comparison**	**Chi-sq (diff)**	***df* (diff)**	***p***
One factor	f1	520.47	90	<0.001	5.78	0.114	[0.106, 0.123]	0.72	–			
Three factors	f3	259.92	87	<0.001	2.99	0.074	[0.065, 0.083]	0.89	m1	192.87	3	<0.001
Revised model	f3r	198.97	85	<0.001	2.34	0.061	[0.051, 0.070]	0.93	m2	49.87	2	<0.001

A model with one common factor (f1) served as the baseline. This model showed poor fit to the data suggesting that one common factor is not enough to describe the covariation between the indicators used. The three-factor model (f3) showed adequate fit to the data based on most criteria (all but CFI) and had a significant improvement in fit compared to f1. However, since several items used almost identical wording and content, a revised model allowing for covariation between the residuals of items 1 (“…*when I want to feel that I am the same person that I was before…”*) and 4 (“…*when I am concerned about whether I am still the same type of person that I was earlier…”)*, and items 8 (“…*when I want to develop more intimacy in a relationship …*”) and 11 (“…when I want to develop a closer relationship with someone.”) was specified (f3r). This model showed adequate fit to the data, and represented a significant improvement of the previous (f3) model. Completely standardized factor loadings obtained with this model are shown in Table 4.

**Table 4:** Factor loadings of the revised model for the three functions of AM.

	**S-C**	**S-B**	**D-B**
Item 1	0.447		
Item 4	0.584		
Item 6	0.760		
Item 13	0.790		
Item 15	0.650		
Item 3		0.681	
Item 8		0.573	
Item 11		0.689	
Item 12		0.636	
Item 14		0.776	
Item 2			0.520
Item 5			0.673
Item 7			0.690
Item 9			0.692
Item 10			0.679

*S-C, self-continuity; S-B, social-bonding; D-B, directing-behavior*.

In pursuit of investigating validity, two sets of multigroup models were specified with the intention of comparing latent means on the three factors between younger and older participants, as well as between males and females. All of these models are shown in Table 5.

**Table 5:** Multigroup comparisons for the revised model for the three functions of AM across age groups and gender.

		**Chi-sq**	***df***	***P***	**NC**	**RMSEA**	**RMSEA 90% CI**	**CFI**	**comparison**	**Chi-sq (diff)**	***df* (diff)**	***P***
**MULTIGROUP (AGE)**												
Configural i.	a1	322.77	170	<0.001	1.89	0.070	[0.059, 0.081]	0.90	–			
Metric i.	a2	330.01	182	<0.001	1.81	0.067	[0.056, 0.077]	0.90	a1	6.97	12	0.86
Scalar i.	a3a	366.13	194	<0.001	1.89	0.070	[0.060, 0.080]	0.89	a2	39.06	12	<0.001
Partial scalar i.	a3b	334.41	185	<0.001	1.81	0.067	[0.056, 0.077]	0.90	a2	4.23	3	0.24
**MULTIGROUP (GENDER)**												
Configural i.	s1	309.14	170	<0.001	1.82	0.067	[0.056, 0.078]	0.91	–			
Metric i.	s2	321.01	182	<0.001	1.76	0.065	[0.054, 0.075]	0.91	s1	11.74	12	0.47
Scalar i.	s3a	344.21	194	<0.001	1.77	0.065	[0.055, 0.075]	0.91	s2	23.57	12	0.023
Partial scalar i.	s3b	326.79	185	<0.001	1.77	0.065	[0.054, 0.075]	0.91	s2	5.85	3	0.12

In both cases models representing configural invariance showed adequate fit to the data, meaning that the same pattern of fixed and free parameters is observed across the two age groups, as well as between genders. In both cases, models representing metric invariance did not have significantly lower fit compared to the configural invariance models. This means that factor loadings for individual items could be treated as equal across the groups. Full scalar invariance, which fixes the intercepts of the items as the same for two groups, failed in both cases. In other words, models constraining item intercepts to be equal across groups showed significantly lower fit compared to the metric invariance models in the case of both age and gender.

Partial scalar invariance (Byrne et al., 1989) requires at least two intercepts per latent factor to be the same across groups, making it a more realistic goal compared to full invariance. In cases of both age and gender, partial scalar invariance was satisfied as evidenced by the lack of decrease of fit between the partial scalar invariance models and the metric invariance models. In case of age, intercepts for items 4 and 13 were constrained across groups for the *Self-Continuity Function* factor, intercepts for items 8 and 11 for the *Social-Bonding Function* factor and intercepts for items 5 and 10 for the *Directing-Behavior Function* factor. Latent means for the younger group were fixed at zero, making them the reference group. In comparison, older participants showed significantly lower value on the *Social-Bonding Function* factor (−0.35, *p* = 0.001) and *Directing-Behavior Function* factor (−0.17, *p* = 0.007). In case of gender, intercepts for the same items as in the case of age were constrained to be equal across groups. Here, males were treated as a reference group and the only difference in the latent means was found for the *Directing-Behavior Function* factor (0.13, *p* = 0.040) indicating that females have higher values than males.

In short, three-factor model of TALE was confirmed in each subsample (men, women, young and older adults). Additionally, differences in the means between these subsamples were found—younger adults scored higher than older adults on the *Social-Bonding Function* and *Directing-Behavior Function* factor, and women scored higher than men on *Directing-Behavior Function* factor.

To gain further evidence in support of the validity of TALE we investigated correlation between three function of AM and related constructs: Self-Concept Clarity, two dimensions of attachment (Anxiety and Avoidance) and two dimensions of past time perspective (Past Positive, Past Negative). All these scales were modeled as single indicator latent variables. To ensure model identification, their error variances were fixed in accordance with their estimated reliabilities and variances. The resulting parameters are presented in Table 6.

**Table 6:** Differential relationships between the three functions of AM and Self-concept clarity, attachment dimensions and time perspectives (Bonferroni's correction applied).

	**Self-clarity**	**Avoidance**	**Anxiety**	**Past positive**	**Past negative**
Self-continuity	−0.386^**^	0.191	0.309^**^	0.167	0.426^**^
Social-bonding	−0.292^**^	−0.035	0.331^**^	0.153	0.320^**^
Directing-behavior	−0.171	0.003	0.100	0.160	0.300^**^

* p < 0.05;

** *p < 0.01*.

## Discussion

Although theorists have long stated the importance of functional approach to AM (e.g., Neisser, 1978; Baddeley, 1988), only recently have empirical attempts begun to shape this approach. Three major theorized functions of AM are its self, social and directive function and Bluck and Alea (2011) have introduced a brief measure of assessing these functions—Thinking About Life Experiences questionnaire (TALE). Although these functions are likely to be universal, gender and country variations could be expected. The current study presents further validation of TALE, in which factorial equivalence across age and gender groups was tested, and investigates the relation between one's tendency to think and talk about the past, and measures of time orientation, self-concept clarity and dimensions of attachment. Since different cultures may vary in terms of psychological values and psychosocial indicators that define everyday context, i.e., in reasons behind the use of past in daily life (Alea et al., 2015), factor structure of TALE was tested in independent samples of older and younger women and men from different cultural setting than in previous studies.

The TALE was developed using CFA and this approach was used in the present study, as well. Previous work has suggested two competing models of overall usage of AM: (i) a theoretical three-factor model consistent with the assumed directive, self and social functions (Pillemer, 1992; Bluck and Alea, 2011) and, (ii) a four-factor model with two social function differentiating between nurturing of existent relationships vs. the development of new ones (Rasmussen and Habermas, 2011; Olivares, 2012). Empirical support of the tripartite model stems from studies conducted in United States (Bluck and Alea, 2011), Trinidad and Tobago (Alea et al., 2015), Japan (Maki et al., 2015). The analysis of the present data provided further empirical support for the tripartite model of AM functions. Factorial equivalence of across age and gender was confirmed. Further favoring the tripartite model of AM functions, the fit indices of this model exceeded fit criteria and the data fit the model well. Accordingly, previous findings have also supported the notion of TALE as a meaningful tool in investigating adult age differences in the functional use of AM (Bluck and Alea, 2008, 2009, 2011).

### Age and gender differences in the use of AM

With regard to age variations, we found that young tend to use AM more for social-bonding and directing purposes. Life span psychologists (Baltes et al., 2016) discuss how specific life phases define one's goals and preoccupations, and how these aspirations relate to using information of what happened in the past to serve one's self, social and directive function. Our findings suggest a somewhat different pattern of using AM in young and old than showed by previous studies. Bluck and Alea (2009) have found that younger adult report more frequent use of AM to create self-continuity and direct behavior, while no age difference was found in using AM for the purpose of social-bonding. Similarly, we found higher use of AM to direct behavior in younger participants. Directive function involves using AM to guide current problem-solving, future behavior, and emotional regulation. Young adults face many important developmental decisions for the first time (e.g., finding work, employment, partners, etc.) and have less, if any, experience in solving everyday existential problems. The future ahead of them seems longer and, therefore, they need more guidance or direction for which they turn to their past experiences (Bluck and Alea, 2009). Consequently, young adulthood is a period in which emotions are intense, yet their expression is more and more required to comply to social norms (Kessler and Staudinger, 2009). Hence, young adulthood calls for stronger and adequate emotion regulation and young adults often turn to their AM to help regulate emotion, which is also considered a directive function of AM (Bluck et al., 2005).

As for age difference in other two functions, self-continuity and social bonding, we found differences in comparison to previous findings. The manner by which AM serves social (bonding) function is by using autobiographical memories for communicational purposes and maintenance of relationships, and it is suggested to be used by young and old alike—young people have to develop significant relationships, while older are guided with the need to maintain and nurture the already developed relationships (Rasmussen and Habermas, 2011). Yet, we found higher use of AM to serve the social-bonding function in young adults. Although opposing previous research, this finding can be seemingly easy explained by the content analysis of items included in the social-bonding subscale of TALE. Namely, most of the items can first handedly be understood as getting to know someone better—e.g., “find out what someone is like” (item 3), “hope to learn more about another person's life” (item 14)—and older adults have presumably already found out and learned a great deal about the life's of their important others. Undoubtedly, maintaining social bonds is important throughout the lifespan (Carstensen, 1993). However, developing new in-depth relations might be more related to younger age. However, Bluck and Alea (2009) did not find an effect of age on the social-bonding function. It is difficult to say whether these different findings stem from cultural differences and different trajectories by which social bonding promotes social identity in different cultures. This issue certainly calls for future investigation.

Finally, we found that both, young and old, are equally prone to use AM to serve their self-continuity. This function is concerned with the question of identity (who I am now, if and how I have changed, and how I have stayed the same over time). It allows individuals to have and endorse a biographical identity (e.g., McAdams, 2001; Bluck and Alea, 2002) and to be able to maintain a coherent self–concept across an entire lifespan (Cohen, 1998). Therefore, AM is used to assure for the integrated sense of self throughout adulthood. In sum, there seem to be age differences in the use of AM to serve its different functions. More importantly, however, it seems that memories can be used in a manner that is sensitive to the demands of specific life-phases. Again, this finding is different from Bluck and Alea (2009) who found an effect of age on the self-continuity function. A possible explanation might lie in the age of participants. Their study looks at the differences between adolescents (mean age 19) and older adults (mean age 73), whereas our sample covers a similar age range, yet compares younger and older adults (mean age 28 vs. 60 years old). It is not surprising that adolescent, with a less clear self-concept, will use AM more for self-continuity purposes, while adults should have less need to do so (Bluck and Alea, 2008).

Regarding gender differences, we found that women tend to use their memories more in directing their behavior. Pillemer, 2003 work on the frequency of use of AM to fulfill different functions provides indirect support for our findings. Using the Reminiscence Function Scale (RFS; Webster, 1993), he identified that women's narratives contained greater number of specific memories than did men's narratives, and women scored higher on problem-solving RFS factor. This RFS factor could be considered as a doppelganger of the directive function of AM (Webster, 1993; Webster and McCall, 1999; Santamaría et al., 2017). It seems that women are less confident about their decisions and express more doubts about their behavior (Bengtsson et al., 2005; Robinson and Stubberud, 2011). Overall, few studies have dealt with gender differences in functions of AM. Although some differences could be traced, it is advised to situate the discussion of gender differences in the cultural context for gender differences in personal memories are usually consistent with the differences found in self-construal across different cultures (Santamaría et al., 2010).

### Convergent and discriminant validity of TALE

Our theory-driven hypothesis that individuals with low self-concept clarity would use memory more often to serve a self-function, those with higher levels of attachment anxiety would use memory more often to serve a social function, and those past-oriented would use memory more often for directive purpose, were all confirmed. Additionally, we also confirmed the expectation that negative past orientation will be more related to the directive use of AM. However, positive past orientation was not found to be significantly correlated to directing-behavior function. One possible explanation for these findings can be found in the content of items forming past positive orientation. Namely, these items mostly reflect nostalgic and sentimental notion of the past, rather than a reference to past decisions which could be used for directing behavior. Finally, we confirmed that avoidant attachment is not correlated with social bonding function of AM and that attachment dimensions are not related to directive function of AM.

We did not formulate clear hypothesis for some other relations, i.e., we expected to find some low correlations between selected constructs and TALE functions because the three functions are somewhat interconnected in everyday life as suggested by Pillemer (2003). Based on the results of this study it seems that self-continuity and social-bonding are more interrelated because significant correlations were found for the use of both of these AM functions and self-clarity, anxious attachment and past negative orientation. Also, directive function seems to be the narrowest of three functions, focusing only on our decisions on future behaviors and it is connected to Past Negative orientation only.

Looking from the perspective of chosen correlates, Past Negative orientation seems to be the most relevant construct. In search of possible explanation, we turn to research on affect and memory which displays an interesting memory pattern—referred to as memory trade-off—namely, the memory for most of the background context of events seems to be traded in favor of memory for the emotional item (Kensinger et al., 2007; Waring and Kensinger, 2009). Although the trade-off can occur for both positive and negative stimuli, memory for negative information often includes more item-specific details. There appears to be much continuity across the adult lifespan in how affective responses guide memory and enhanced retention of item-specific details of negative information is mostly well preserved across the adult lifespan (Denburg et al., 2003; Kensinger et al., 2007). Obviously, negative experiences and events are stored and preserved with more particularities. Thus, they seem to be a more reliable advisor in situations when past experiences and memories are used, and needed as a reference point for our current and future social bonds, behavior and coherence of our self-image.

### Limitations and future directions

Although results look promising for the tripartite model of AM functions, several methodological issues need to be considered. Firstly, TALE questionnaire has a difficult instruction to follow and we believe it is justified to question the ability of average person to answer in a way that is required (i.e., to state to what extent when remembering the past he/she does so in order to fulfill a specific function). It is more plausible that answers reflect how often people think of the past in a depicted manner. Also, the use of TALE assumes that people are aware of the AM functions and encode the function that specific memory serves when it is recalled, and that they access this information and use it when responding. It seems more plausible that TALE captures people's believes about their uses of AM, which can sometimes be accurate and grounded in experience. However, it could be that responses reflect naïve assumptions about the functions memory should serve and this issue calls for further investigation. Addressing this issue, Maki et al. (2015) have asked participants to answer the TALE questionnaire and to provide specific instances of recalling AM for different functions. They found that the TALE scores correlated on the group level with the proportion of provided instances of AM. Although their study does not show that the TALE measures the use of AM for different functions, it does address this issue and marks the path for further investigation.

Secondly, TALE as a construct does not encompass the whole range of uses of memory in everyday life. It is operationalized through a substantial reliance on tripartite model and, therefore, it is perhaps not surprising that this scale confirms such model. However, unlike TALE, the Reminiscence Functions Scale (Webster, 1993) recognizes eight different functions and recognizes that these functions may vary/change with age. Furthermore, AM functions based on TALE are relatively narrow in their focus. On the level of item content, directive function reflects only directive behaviors, and disregards self-focus and emotion regulation. Social Bonding function focuses mainly on forming new friendships and neglects friendship maintenance. Also, TALE only focuses on the extent to which people remember the past and not the valence of these memories. However, the valence of memories can have different effect on AM functions. For example, we could expect that individuals use more positive than negative AM memories in order to serve social bonding function (Rasmussen and Berntsen, 2009). Finally, although large and heterogeneous in many ways our sample was still a convenient sample. Although our results support the tripartite model of AM functions, and assure for the justified use of TALE in both older and younger women and men, future studies should employ more representative samples, as well as different measures, to investigate further the convergent validity of the TALE questionnaire.

## Author contributions

All authors have contributed equally to all the aspects of research and manuscript preparation.

### Conflict of interest statement

The authors declare that the research was conducted in the absence of any commercial or financial relationships that could be construed as a potential conflict of interest.
